# Supplementary figures and images for: Effective Quality Breeding Directions—Comparison and Conservative Analysis of Hepatic Super-Enhancers between Chinese and Western Pig Breeds
Source: Biology (Basel). 2022 Nov 8;11(11):1631. doi: 10.3390/biology11111631 (PMC9687233; doi:10.3390/biology11111631)

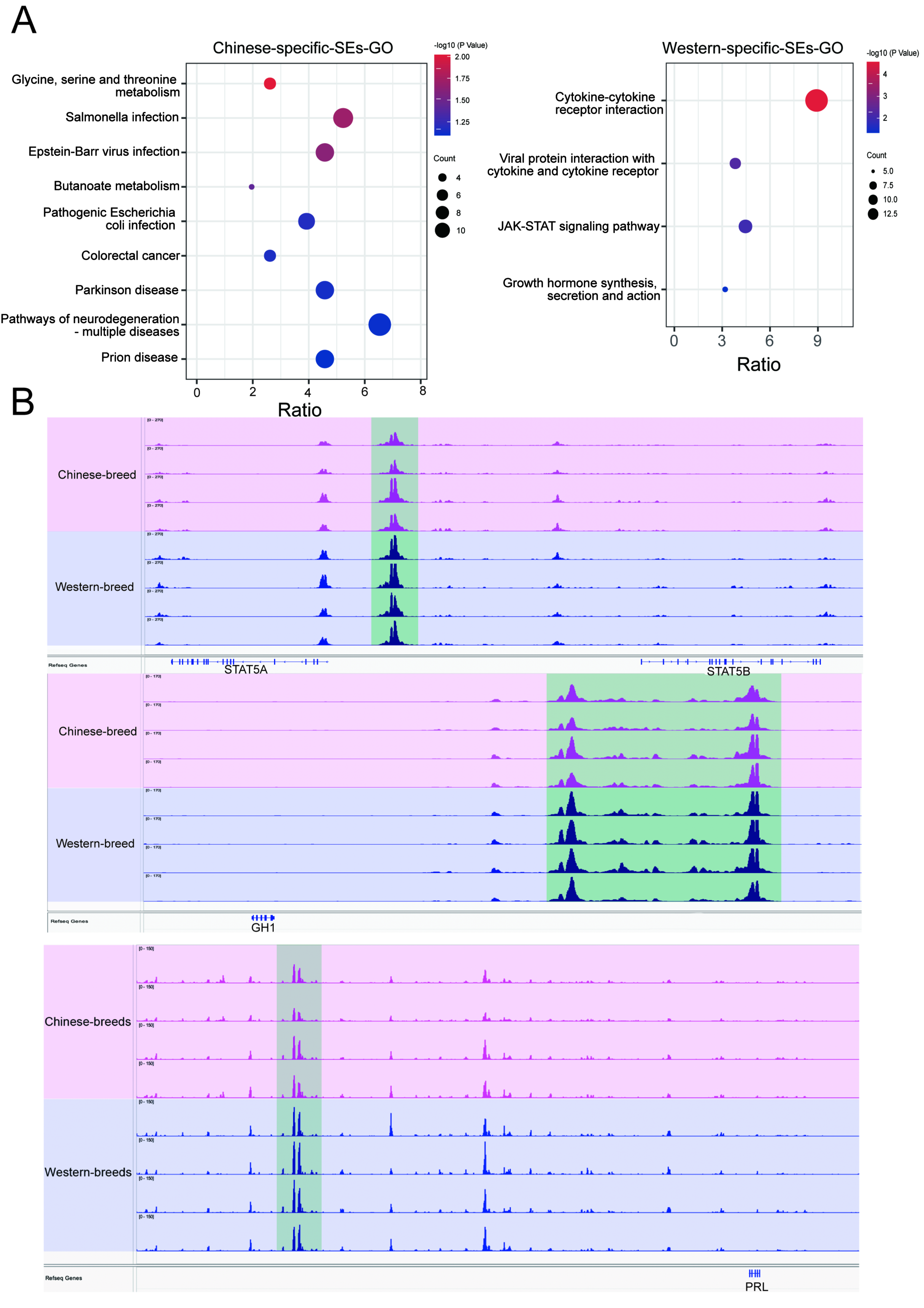

Supplement: Supplementary file 1 [file biology-11-01631-s001.zip › Figure S1.tif]
